# Effect of tocilizumab on haematological markers implicates interleukin-6 signalling in the anaemia of rheumatoid arthritis

**DOI:** 10.1186/ar4397

**Published:** 2013-12-02

**Authors:** John D Isaacs, Olivier Harari, Uwe Kobold, Janet S Lee, Corrado Bernasconi

**Affiliations:** 1 Institute of Cellular Medicine, The Medical School, Framlington Place, Newcastle upon Tyne, NE2 4HH, UK; 2 Roche, Basel, Switzerland; 3 Roche Diagnostics GmbH, Penzberg, Germany; 4 Roche, Nutley, New Jersey; 5 Newcastle University, Institute of Cellular Medicine (Musculoskeletal Research Group), Newcastle upon Tyne, NE2 4HH, UK

## Abstract

**Introduction:**

Our objective was to determine the interrelationships of interleukin (IL)-6 receptor inhibition with haemoglobin, acute-phase reactants and iron metabolism markers (including hepcidin) in patients with rheumatoid arthritis (RA).

**Methods:**

Data of patients receiving tocilizumab or placebo in the MEASURE study were analysed. We investigated associations at baseline and during tocilizumab treatment among haemoglobin, parameters of haemoglobin and iron homeostasis [ferritin, total iron-binding capacity (TIBC), hepcidin, haptoglobin], IL-6 and acute-phase reactants [C-reactive protein (CRP), erythrocyte sedimentation rate (ESR)] to identify statistical correlates of rise in haemoglobin level.

**Results:**

At baseline, CRP and haptoglobin were inversely correlated (modestly) with haemoglobin levels. After treatment with tocilizumab, CRP, hepcidin, ferritin and haptoglobin levels fell alongside increases in TIBC and haemoglobin. The falls in CRP, hepcidin and haptoglobin levels in the first 2 weeks correlated with a week 12 rise in TIBC and haemoglobin.

**Conclusions:**

Inflammatory anaemia improves in patients with RA treated with tocilizumab. This improvement correlates with the degree of suppression of systemic inflammation, reduction in hepcidin and haptoglobin and increase in iron-binding capacity. These clinical data provide evidence of a role for IL-6 signalling in the inflammatory anaemia of RA.

## Introduction

One of the mechanisms of the inflammatory response is the sequestration of iron in macrophages, leading to decreased availability of iron to invading pathogens. Chronic inflammatory diseases—including Castleman’s disease [1], systemic-onset juvenile idiopathic arthritis [2] and rheumatoid arthritis (RA) [3]—are often accompanied by anaemia. Hepcidin acts as a gatekeeper for transmembrane iron transport [3], binding to the cellular iron efflux channel ferroportin, inducing its internalisation and degradation [4]. Thus, hepcidin inhibits intestinal iron absorption and decreases iron mobilisation from macrophage reticuloendothelial stores [3,5]. Ferritin scavenges free iron and enables its sequestration in macrophage reticuloendothelial stores. Haptoglobin decreases iron availability in the setting of haemolysis, rather than inflammation, by scavenging circulating haemoglobin [6]. Transferrin, the principal iron carrier, regulates the total iron-binding capacity (TIBC) of blood. Hepcidin, ferritin and haptoglobin are produced in the liver as acute-phase reactants, and their expression is dependent on interleukin-6 (IL-6) signalling [3,7,8]. Tocilizumab is a humanised monoclonal antibody that inhibits IL-6 binding to its receptor [9].

Tocilizumab reduces hepcidin levels and improves anaemia in patients with Castleman’s disease [1] and in an animal model of arthritis [10]. Tocilizumab treatment is also associated with increased haemoglobin levels in patients with RA [11-15]. The current analysis tested associations between changes in haematological parameters and acute-phase markers in a phase 3B clinical trial of tocilizumab in RA (MEASURE) [16,17].

## Methods

### Patients

This was an exploratory *post hoc* analysis of data from MEASURE, a randomised, multicentre, double-blind, 24-week, phase 3B trial with an open-label follow-up period of two years. Participants were 18 years of age or older and had moderate to severe active RA of more than six months duration and inadequate responses to methotrexate (MTX). They had ≥6 swollen and tender joints, with either C-reactive protein (CRP) >10 mg/L or erythrocyte sedimentation rate (ESR) ≥28 mm/hour at screening and were randomly assigned 1:1 to receive tocilizumab 8 mg/kg intravenously (IV) or placebo every four weeks plus weekly MTX. For the current analysis, patients were analysed according to the treatment they received during the first 12 weeks of the study (that is, before rescue therapy was permitted per protocol). The study was approved by independent ethics committees outside the United States (approval was obtained from a local review board; if there was no local review board, approval was obtained from a regional committee; if there was no regional committee, approval was obtained from the European Ethics Review Committee) and institutional review boards in the United States, and all subjects consented in writing to participation. A full listing of ethics committees and institutional review boards is available in Additional file 1: Table S1.

### Assessments

Serum hepcidin was measured by high-performance liquid chromatography–tandem mass spectroscopy assay [18]. Serum haptoglobin was determined using a Roche Diagnostic immunoturbidimetric assay kit. Anti-haptoglobin antibodies mixed with sample produced immunocomplexes that were quantified using the Roche Modular P autoanalyser (Roche Diagnostics, Mannheim, Germany) and detection at 340 nm. Markers of inflammation and iron homeostasis were measured using standard available assays. Assessments were performed at screening, baseline, day 1 and weeks 1, 2, 4, 8, 12 and 24 during randomised treatment.

### Statistical analysis

Descriptive statistical methods and univariate and multivariate linear regression were used. Specific regression models were refined using a statistical selection procedure: successive variable elimination based on Akaike’s Information Criterion. Because of the distribution of raw values, logarithmic transformation was used for high-sensitivity CRP, hepcidin and ferritin values in statistical models. In addition, in correlation analyses, Pearson’s coefficient was complemented with the non-parametric Spearman’s rank correlation.

## Results

### Patients

The study enrolled 132 patients; 63 were randomly assigned to placebo + MTX (control) and 69 to tocilizumab + MTX. One patient assigned to placebo received tocilizumab from study start and was evaluated in the tocilizumab group for the current analysis. Mean age was slightly higher in this study than in the tocilizumab phase 3 pivotal trials; however, disease characteristics were similar [11-15]. Baseline demographics and disease characteristics were generally similar between patients in the tocilizumab and the control groups (Additional file 2: Table S2). The median age of patients in both groups was 57.0 years. A slightly higher proportion of patients in the tocilizumab group were women (83% and 74%, respectively), and they had slightly higher CRP at baseline (median, 12.5 and 10.4 mg/L). The median duration of RA at baseline was similar between groups (7.0 years (tocilizumab) versus 6.8 years (control)), as were Disease Activity Scores using 28 joints (DAS28) (6.8 (tocilizumab) versus 6.6 (control)). Overall, 7.1% of tocilizumab and 11.3% of placebo patients had anaemia at baseline by World Health Organization criteria (haemoglobin: <11 g/dl (women), <12 g/dl (men)).

### Baseline parameters and correlations

Haemoglobin levels at baseline exhibited modest inverse correlations with the acute-phase reactants CRP and haptoglobin (Spearman rank correlation [ρ] = -0.20 and -0.25, respectively; *P* = 0.023 and *P* = 0.004, respectively) and only minimal correlation with IL-6 (Additional file 3: Figure S1). Pretreatment hepcidin was highly variable (0 to 63.1 fmol/μl; coefficient of variation (CV) 1.2) (Additional file 4: Figure S2). CVs were lower for ferritin (0.86), haptoglobin (0.43) and TIBC (0.17). Haemoglobin did not correlate with hepcidin (Additional file 5: Figure S3) or TIBC. Ferritin and haemoglobin levels had a weak positive association, with the effect more pronounced at very low ferritin levels. There was also a trend towards low mean corpuscular volume (MCV) with low ferritin. Hepcidin levels significantly correlated with haemoglobin homeostasis markers ferritin (ρ = 0.64) and TIBC (inversely; ρ = -0.34) and also with IL-6, CRP and haptoglobin (ρ = 0.34-0.46; *P* ≤0.001) (Additional file 5: Figure S3).

### Longitudinal effect of tocilizumab

**Figure 1:**
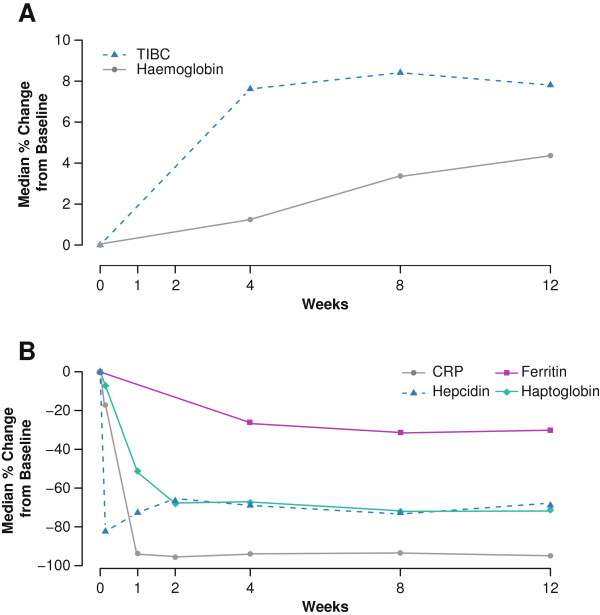
**Time course of median percentage change in (A) haematological and (B) inflammatory markers.** Patients received tocilizumab + MTX. TIBC, total iron-binding capacity. MTX, methotrexate.

Increases in haemoglobin levels and early reductions in hepcidin, CRP, haptoglobin and ESR were observed in association with tocilizumab therapy (n = 70) (Figure 1). A rapid decline in hepcidin level was apparent from day 1 (*P* <0.001 versus placebo). Declines in ESR (not shown), CRP and haptoglobin levels were detectable by week 1. Haemoglobin levels increased by week 4 and increased further to week 12, reaching a plateau by week 24 (data not shown). Ferritin levels fell and TIBC increased with kinetics similar to those of haemoglobin (Figure 1).

### Longitudinal relationships

**Figure 2:**
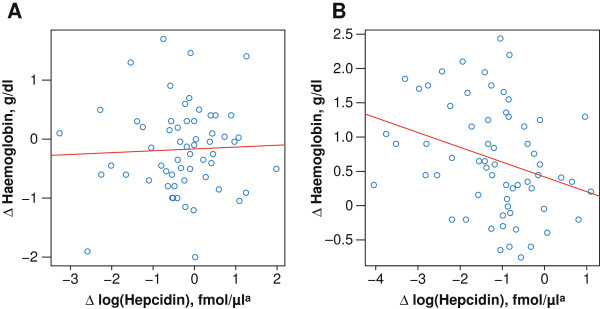
**Scatter plot and linear regression of haemoglobin level at week 12 versus log hepcidin.** Change from baseline in haemoglobin level (g/dl) at week 12 versus mean of change from baseline at weeks 1 and 2 in log hepcidin (fmol/μL). **(A)** Placebo: *r*^2^ = 0.002; *P* = 0.76; Spearman rank correlation: ρ = 0.04, *P* = 0.79. **(B)** Tocilizumab: *r*^2^ = 0.09; *P* = 0.012; Spearman rank correlation: ρ = -0.35, *P* = 0.005. ^a^Mean of weeks 1 and 2 assessments.

In tocilizumab-treated patients, an early decline in hepcidin level (mean of decline at weeks 1 and 2 for each patient) correlated with a subsequent increase in haemoglobin level (at weeks 4, 8 and 12) (ρ >0.31 and *P* <0.011 for all comparisons) (Figure 2). These effects were not evident for the placebo group, as indicated in Figure 2 and by a significant interaction between treatment group assignment and hepcidin change (*P* = 0.023) in models of the haemoglobin change adjusted for baseline haemoglobin. Therefore, further evaluations of treatment-related longitudinal effects were performed in the tocilizumab group only. Furthermore, a decline in CRP and haptoglobin level at week 2 correlated with a week 12 increase in haemoglobin level (ρ >0.33 and *P* <0.026 for all comparisons) (Additional file 6: Figure S4). In multivariate analyses, patient-to-patient variability in haemoglobin increase through week 12 remained largely unexplained, but the strongest association was with a fall in haptoglobin (multiple *r*^2^ = 0.29; *P* <0.0033) (Table 1). This effect remained evident in analyses limited to patients with baseline haemoglobin levels below the median (multiple *r*^2^ = 0.49; *P* = 0.0018).

**Table 1:** Multivariate analysis of tocilizumab-induced increases in haemoglobin at week 12

**Statistical model**	**Coefficient**	**SE**	***P***
**Basic linear model in the tocilizumab arm**^**a**^			
Intercept	2.20	1.93	0.26
Baseline haemoglobin, g/dl	-0.19	0.14	0.20
Change log(CRP) baseline, week 2	-0.048	0.15	0.75
log(Hepcidin) change from baseline to week 2, fmol/μl	-0.044	0.11	0.69
Haptoglobin change from baseline to week 2, fmol/μl	-0.0052	0.0028	0.076
Male	0.042	0.45	0.93
**Refined multivariate model**^**b**^			
Intercept	2.20	1.68	0.20
Baseline haemoglobin, g/dl	-0.18	0.12	0.15
log(Haptoglobin) change from baseline to week 2, fmol/μl	-0.0060	0.0021	0.0079

^a^Multiple *r*^2^ = 0.30, *P* = 0.047; ^b^Multiple *r*^2^ = 0.29, *P* < 0.0033. CRP, C-reactive protein; SE, standard error.

## Discussion

The iron-sequestering actions of hepcidin are hypothesised to be the major determinant of inflammatory anaemia [3,19]. In support of this mechanistic understanding, tocilizumab induced rapid and sustained decreases in hepcidin that were associated with subsequent increases in haemoglobin. At baseline, hepcidin levels were inversely correlated with TIBC, underscoring the role of hepcidin in reducing iron bioavailability. Ferritin, hepcidin and haptoglobin at baseline correlated with ESR, CRP and IL-6 (Additional file 5: Figure S3). This underscores their behaviour as acute-phase reactants, as does the correlation between ferritin and hepcidin. The rapid declines in their levels after tocilizumab treatment were consistent with IL-6 signalling being a common stimulus to their production. At baseline, there was a weak inverse correlation between haemoglobin and the acute-phase reactants but no association with hepcidin. This may reflect the high variability in baseline hepcidin.

Nevertheless, this underscores the current understanding of the relationship between hepcidin and haemoglobin as an indirect one, mediated through the regulation of iron transport. Tocilizumab had an immediate and profound effect on hepcidin levels, whereas its effect on haemoglobin was slower; the acute-phase reactants—haptoglobin, in particular—showed an intermediate kinetic. The more similar kinetics of haptoglobin and haemoglobin changes might have strengthened their association compared with those of hepcidin and haemoglobin. However, because this was a *post hoc* analysis of clinical trial data, the ability to assess causal relationships is limited. This is a limitation of the study, which focuses on the strength of associations but cannot elucidate the structure of the interaction network. Although reductions in hepcidin clearly influence subsequent increases in haemoglobin level, we cannot exclude other influences on haemoglobin levels. The observation that a small subset of patients with the lowest ferritin levels also had low haemoglobin levels and low MCV indicates that in these patients, iron deficiency might have been driving anaemia more than inflammation. However, the number of such patients appears too small to have impacted the relationship between tocilizumab treatment and resolution of inflammatory anaemia evident in the overall population. The MEASURE study population had a relatively modest baseline CRP elevation in spite of moderate to high DAS28 scores, so it is possible that the associations reported here would have been more marked in a cohort of patients with higher levels of systemic inflammation and lower levels of haemoglobin. Patients assigned to tocilizumab had slightly higher CRP levels and DAS28 scores but also slightly higher haemoglobin levels than those assigned to placebo. Although the differences were not great enough to affect the results, the counterintuitive directionality of inflammation versus anaemia underscores the variability associated with these parameters, even in a study of more than 100 patients.

The overall pattern of normalisation in haematological parameters engendered by tocilizumab is in keeping with previous reports with this therapy in Castleman’s disease [1] and strongly supports a role for IL-6 in the inflammatory anaemia of RA. To dissect the influence of various downstream pathways, however, would require an appropriately powered study specifically in anaemic RA patients, with a purposely designed sampling scheme and an examination of the influence of other therapies.

## Conclusions

In patients with RA treated with tocilizumab, reductions in inhibitors of iron transport and availability, hepcidin and haptoglobin, as well as the acute-phase reactant CRP, were associated with subsequent increases in haemoglobin. Therefore, inflammatory anaemia in RA appears to involve IL-6 signalling and improves with tocilizumab treatment.

## Abbreviations

CRP: C-reactive protein; CV: Coefficient of variation; DAS28: Disease activity score using 28 joints; ESR: Erythrocyte sedimentation rate; IL-6: Interleukin-6; IV: Intravenously; MCV: Mean corpuscular volume; MTX: Methotrexate; RA: Rheumatoid arthritis; SE: Standard error; TIBC: Total iron-binding capacity.

## Competing interests

JDI has received an unrestricted research grant, consultancy fees and lecture/speaker honoraria from F. Hoffmann-La Roche. OH is a former employee of and a stockholder in Roche Products Ltd. Funding for manuscript preparation was provided by F. Hoffmann-La Roche Ltd. CB is a biostatistician contractor for F. Hoffmann-La Roche. UK has no competing interests. JSL is a former employee of and a stockholder in Roche.

## Authors’ contributions

JDI conceived of and designed the study, acquired data, analysed and interpreted the data, drafted the manuscript and revised it critically for important intellectual content. OH conceived of and designed the study, acquired data, analysed and interpreted the data, drafted the manuscript and revised it critically for important intellectual content. All authors read and approved the final manuscript. CB analysed and interpreted the data, drafted the manuscript and revised it critically for important intellectual content. UK developed the HPLC-MS/MS method for hepcidin measurement and reviewed the manuscript for important intellectual content. JSL conceived of and designed the study, acquired data, analysed and interpreted the data and drafted the manuscript and revised it critically for important intellectual content.

## Supplementary Material

Additional file 1: Table S1 Listing of ethics committees and institutional review boards that approved the study. Click here for file

Additional file 2: Table S2 Baseline demographics, disease and haematologic factors. Click here for file

Additional file 3: Figure S1 Scatter plot and linear regression for baseline haemoglobin versus log baseline CRP, haptoglobin and IL-6. **(A)** Baseline haemoglobin versus log baseline CRP (mg/dl). **(B)** Baseline haemoglobin versus baseline haptoglobin (mg/dl). **(C)** Baseline haemoglobin versus log baseline IL-6 (pg/ml). **(A)***r*^2^ = -0.18, *P* = 0.039; Spearman rank correlation: ρ = -0.20, *P* = 0.023. **(B)***r* = -0.27, *P* = 0.0015; Spearman rank correlation: ρ = -0.25, *P* = 0.004. **(C)***r*^2^ = -0.15, *P* = 0.15, Spearman rank correlation: ρ = -0.19, *P* = 0.048. CRP, C-reactive protein. Click here for file

Additional file 4: Figure S2 Levels of hepcidin (fmol/μl). **(A)** Baseline. **(B)** Week 1. Click here for file

Additional file 5: Figure S3 Scatter plots and linear regression of baseline hepcidin versus baseline ferritin, CRP, haptoglobin and haemoglobin. Baseline hepcidin versus baseline **(A)** ferritin, **(B)** CRP, **(C)** haptoglobin and **(D)** haemoglobin levels. (A) *r*^2^ = 0.47, ρ = 0.64, *P* <0.0001. (B) *r*^2^ = 0.11, ρ = 0.34, *P* = 0.0001. (C) *r*^2^ = 0.24, ρ = 0.45, *P* <0.0001. (D) *r*^2^ = 0.004, ρ = 0.34, *P* <0.49. Click here for file

Additional file 6: Figure S4 Scatter plot and linear regression of change from baseline in haemoglobin level (g/dl). Change from baseline in haemoglobin level (g/dl) at week 12 versus mean of change from baseline at weeks 1 and 2 in log CRP (mg/dl) and haptoglobin (mg/dl). **(A)***r*^2^ = -0.31, *P* = 0.040; ρ = -0.33, *P* = 0.026. **(B)***r*^2^ = -0.44, *P* <0.001; ρ = -0.43, *P* <0.001. Click here for file
