# Supplementary material for: Effect of tocilizumab on haematological markers implicates interleukin-6 signalling in the anaemia of rheumatoid arthritis
Source: Arthritis Res Ther. 2013 Dec 2;15(6):R204. doi: 10.1186/ar4397 (PMC3978585; doi:10.1186/ar4397)
Supplement: Additional file 1: Table S1 — Listing of ethics committees and institutional review boards that approved the study. [file ar4397-S1.doc]

**Additional file 1: Table S**1 Listing of ethics committees and institutional review boards that approved the study

| **Ethics committee/institutional review board** | **Country** |
| --- | --- |
| Newcastle and North Tyneside Research Ethics Committee #02 | United Kingdom |
| North West Multi-centre Research Ethics Committee | United Kingdom |
| Greater Glasgow and Clyde Research and Development Office | United Kingdom |
| IRB Services | Canada |
| Health Research Ethics Board | Canada |
| Quorum Review | United States |
| Lehigh Valley Hospital Institutional Review Board | United States |
| Office of Human Subjects Research – Johns Hopkins Medicine Institutional Review Boards | United States |
