# Supplementary material for: Effect of tocilizumab on haematological markers implicates interleukin-6 signalling in the anaemia of rheumatoid arthritis
Source: Arthritis Res Ther. 2013 Dec 2;15(6):R204. doi: 10.1186/ar4397 (PMC3978585; doi:10.1186/ar4397)
Supplement: Additional file 2: Table S2 — Baseline demographics, disease and haematologic factors. [file ar4397-S2.doc]

**Additional file 2: Table S2** Baseline demographics, disease and haematologic factors

|  | **Placebo + MTX**  **n = 62** | **Tocilizumab + MTX**  **n = 70** |
| --- | --- | --- |
| Women, n (%) | 46 (74) | 58 (83) |
| Age, years (range) | 56.5 (50.0-64.0) | 57.0 (49.0-62.0) |
| Weight, kg (range) | 82.0 (65.0-92.1) | 77.2 (67.0-86.5) |
| BMI, kg/m^2^, median (range) | 29.3 (18.5-49.6) | 29.2 (19.4-57.3) |
| Current smoker, n (%) | 14 (23) | 19 (27) |
| History of diabetes, n (%) | 4 (6) | 6 (9) |
| Duration of RA, years (range) | 6.8 (2.4-9.9) | 6.8 (2.0-16.2) |
| DAS28 (range) | 6.6 (5.8-7.2) | 6.8 (5.9-7.4) |
| CRP, mg/dl (range) | 0.88 (0.39-1.86) | 0.93 (0.52-2.65) |
| Statin use, n (%) | 10 (16) | 10 (14) |
| Previous aTNF, n (%) | 20 (32) | 28 (40) |
| Oral steroid use, n (%) | 16 (26) | 21 (30) |
| Baseline MTX dose, mg/week (range) | 15.0 (15.0-20.0) | 15.0 (15.0-20.0) |
| Haemoglobin, g/dL (range) | 12.7 (11.9-13.3) | 12.9 (11.9-13.5) |
| Ferritin, ng/mL (range) | 74.2 (36.0-135.1) | 77.3 (36.9-123.1) |
| TIBC, μg/dL (range) | 280.0 (260.3-316.4) | 284.3 (249.0-311.3) |
| Haptoglobin, mg/dL (range) | 193.0 (140.0-265.0) | 216.5 (163.3-270.5) |
| Hepcidin, fmol/μL (range) | 4.2 (1.6-10.2) | 6.3 (1.7-10.1) |
| IL-6, pg/mL (range) | 19.4 (6.2-47.8) | 13.8 (6.1-32.1) |

aTNF, anti–tumour necrosis factor; BMI, body mass index; CRP, C-reactive protein; DAS28,

Disease Activity Score using 28 joints; IL-6, interleukin-6; MTX, methotrexate; RA, rheumatoid arthritis; TIBC, total iron-binding capacity.

Data are presented as median (interquartile range) unless otherwise indicated.
