# Supplementary figures and images for: Effect of tocilizumab on haematological markers implicates interleukin-6 signalling in the anaemia of rheumatoid arthritis
Source: Arthritis Res Ther. 2013 Dec 2;15(6):R204. doi: 10.1186/ar4397 (PMC3978585; doi:10.1186/ar4397)

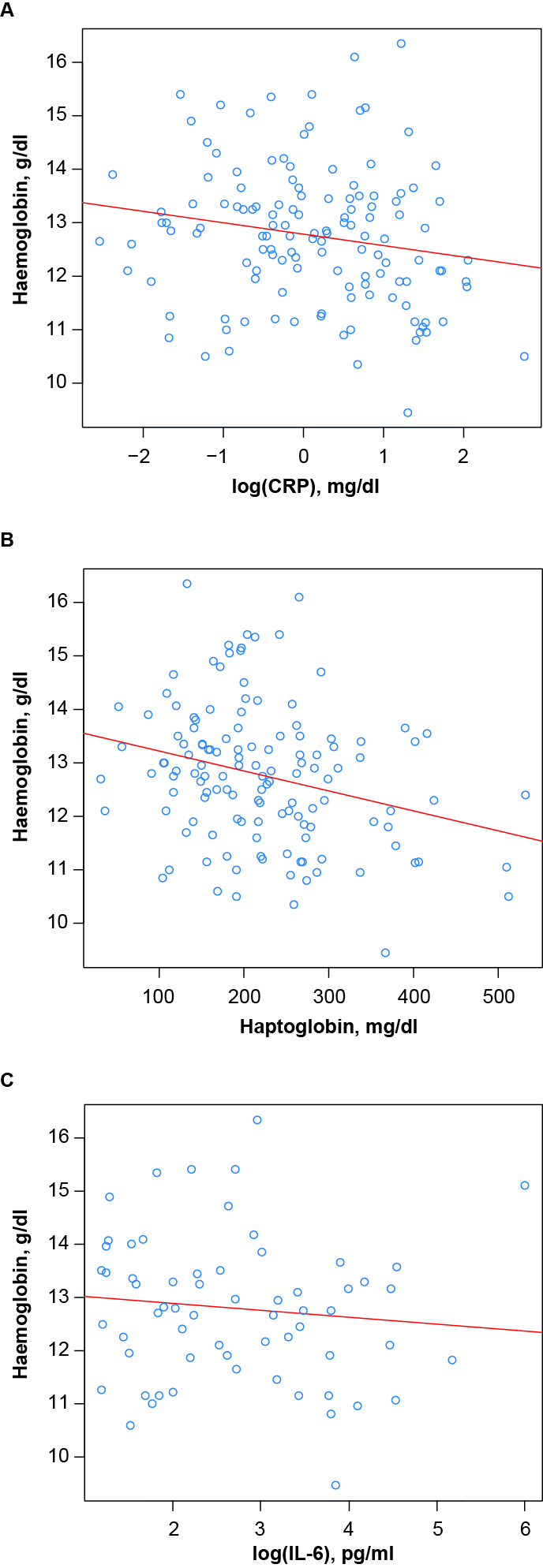

Supplement: Additional file 3: Figure S1 — Scatter plot and linear regression for baseline haemoglobin versus log baseline CRP, haptoglobin and IL-6. (A) Baseline haemoglobin versus log baseline CRP (mg/dl). (B) Baseline haemoglobin versus baseline haptoglobin (mg/dl). (C) Baseline haemoglobin versus log baseline IL-6 (pg/ml). (A)r2 = -0.18, P = 0.039; Spearman rank correlation: ρ = -0.20, P = 0.023. (B)r = -0.27, P = 0.0015; Spearman rank correlation: ρ = -0.25, P = 0.004. (C)r2 = -0.15, P = 0.15, Spearman rank correlation: ρ = -0.19, P = 0.048. CRP, C-reactive protein. [file ar4397-S3.doc]

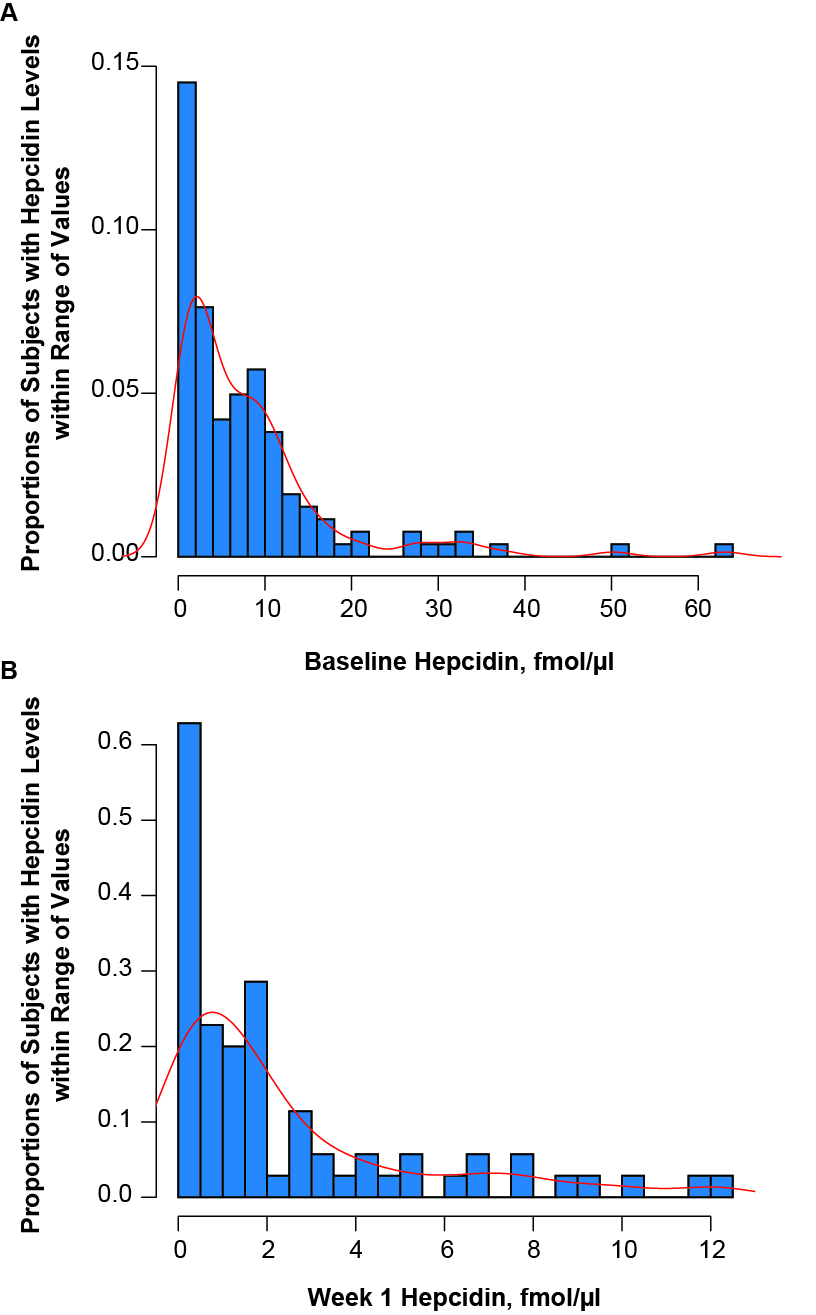

Supplement: Additional file 4: Figure S2 — Levels of hepcidin (fmol/μl). (A) Baseline. (B) Week 1. [file ar4397-S4.doc]

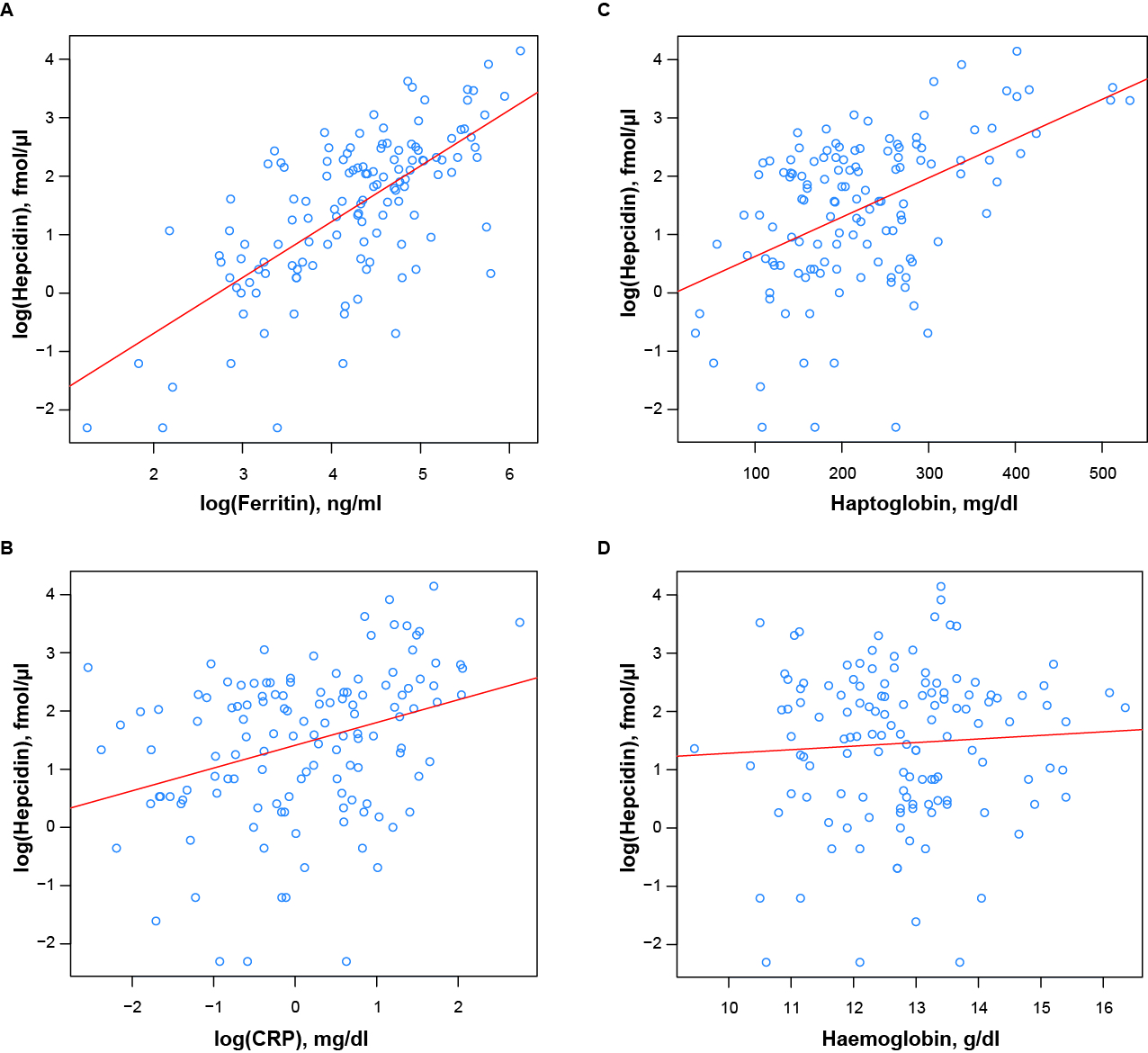

Supplement: Additional file 5: Figure S3 — Scatter plots and linear regression of baseline hepcidin versus baseline ferritin, CRP, haptoglobin and haemoglobin. Baseline hepcidin versus baseline (A) ferritin, (B) CRP, (C) haptoglobin and (D) haemoglobin levels. (A) r2 = 0.47, ρ = 0.64, P <0.0001. (B) r2 = 0.11, ρ = 0.34, P = 0.0001. (C) r2 = 0.24, ρ = 0.45, P <0.0001. (D) r2 = 0.004, ρ = 0.34, P <0.49. [file ar4397-S5.doc]

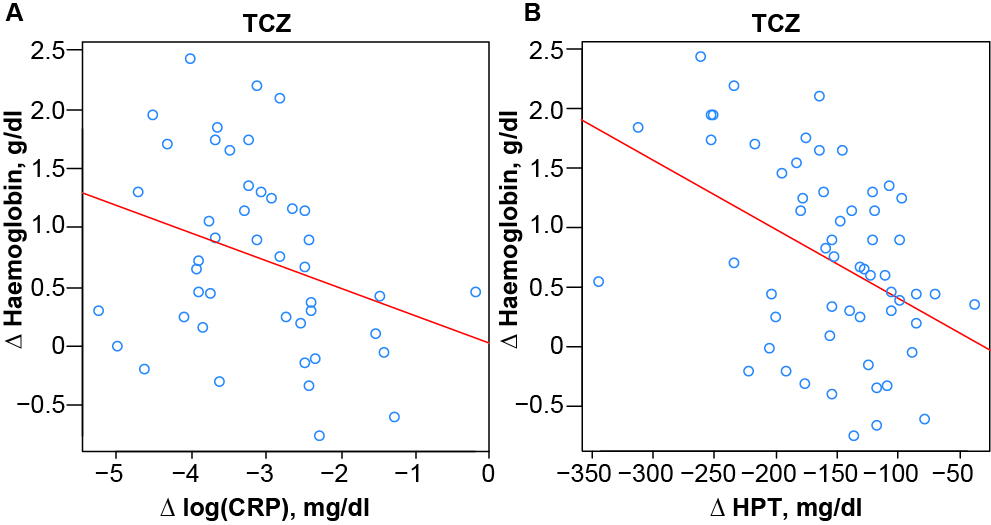

Supplement: Additional file 6: Figure S4 — Scatter plot and linear regression of change from baseline in haemoglobin level (g/dl). Change from baseline in haemoglobin level (g/dl) at week 12 versus mean of change from baseline at weeks 1 and 2 in log CRP (mg/dl) and haptoglobin (mg/dl). (A)r2 = -0.31, P = 0.040; ρ = -0.33, P = 0.026. (B)r2 = -0.44, P <0.001; ρ = -0.43, P <0.001. [file ar4397-S6.doc]
